# Grape skin extract modulates neuronal stem cell proliferation and improves spatial learning in senescence-accelerated prone 8 mice

**DOI:** 10.18632/aging.203373

**Published:** 2021-07-28

**Authors:** Kazunori Sasaki, Noelia Geribaldi-Doldan, Francis G. Szele, Hiroko Isoda

**Affiliations:** 1 Alliance for Research on the Mediterranean and North Africa (ARENA), University of Tsukuba, Tsukuba, Ibaraki 305-8572, Japan; 2 Open Innovation Laboratory for Food and Medicinal Resource Engineering, National Institute of Advanced Industrial Science and Technology (AIST) and University of Tsukuba, Tsukuba, Ibaraki 305-8572, Japan; 3 Department of Physiology, Anatomy and Genetics, University of Oxford, Oxford OX1 3QX, UK; 4 Faculty of Life and Environmental Sciences, University of Tsukuba, Tsukuba, Ibaraki 305-8572, Japan

**Keywords:** grape skin, neuroprotection, neuronal stem cell, SAMP8, neuroinflammation

## Abstract

In recent years, the number of patients with neurodegenerative illness such as Alzheimer’s disease (AD) has increased with the aging of the population. In this study, we evaluated the effect of Grape skin extract (GSE) on neurotypic SH-SY5Y cells as an *in vitro* AD model, murine neurospheres as an *ex vivo* neurogenesis model and SAMP8 mice as an *in vivo* AD model. Our *in vitro* result showed that pre-treatment of SH-SY5Y cells with GSE ameliorated Aβ-induced cytotoxicity. Moreover, GSE treatment significantly decreased the number of neurospheres, but increased their size suggesting reduced stem cell self-renewal but increased proliferation. Our *in vivo* Morris water maze test indicated that GSE improves learning and memory in SAMP8 mice. To detect proliferation and newborn neurons, we measured BrdU+ cells in the dentate gyrus (DG). GSE treatment increased the number of BrdU+ cells in the DG of SAMP8 mice. Finally, we showed that GSE induced a decrease in inflammatory cytokines and an increase in neurotransmitters in the cerebral cortex of SAMP8 mice. These results suggested that GSE increased neurogenic zone proliferation and memory but decreased oxidative stress associated with pro-inflammatory cytokines in aging, thus protecting neurons.

## INTRODUCTION

The grape (Vitis vinifera) is one of the most cultivated fruit crops in the world and one of the chief dietary sources of polyphenols and flavonoids [1]. Flavonoids, a type of polyphenol, have been postulated as dietary supplements that can exhibit neuroprotection and neuronal regeneration [2, 3] and also prevent age-related neurodegeneration [4, 5]. Anthocyanins are among the most important dietary flavonoids and more than 635 anthocyanins have been identified, including delphinidin and malvidin two of the most studied [6]. Anthocyanins are mainly localized in the grape’s skin [7], where they are found at concentrations of 200 to 5,000 mg/kg of fresh grapes [8]. Anthocyanins are important in common diets because, in addition to grapes, they are present in other fruits and vegetables, and also in beverages such as tea, fruit juice, cocoa and wine [9, 10]. Several studies have demonstrated that dietary anthocyanins can be beneficial in a variety of ways. Anthocyanins have anti-cancer activity [11], anti-oxidant activity [12], anti-obesity activity [13], and anti-diabetic activity [14]. Also, they were anti-inflammatory in several diseases [15, 16]. Some flavonoids can cross the blood brain barrier [17] suggesting they may have beneficial effects in the CNS and positioning them for the development of new drugs to prevent neurodegenerative diseases. Flavonoids exert neuroprotective effects on the brain and prevent age-related neurodegeneration via prevention of oxidative stress [18] and Aβ-induced neuronal death [19]. Also, flavonoids improve memory and cognitive deficits associated with the ageing process [20, 21]. Other polyphenols such Resveratrol, which is also found in grape skin increased net neurogenesis in the hippocampus of aged rats and prevented cognitive decline [22]. Resveratrol also enhanced the microvasculature of the hippocampus and reduced hypertrophy of astrocytes [22].

Neurogenesis occurs in the adult brain in the dentate gyrus (DG) of the hippocampus [23, 24]. The DG generates neurons important for memory and stress [25]. Alzheimer’s disease (AD) is characterized by memory loss and also progressive cognitive impairment, deposits of amyloid-beta (Aβ), neurofibrillary tangles, and loss of neurons and synapses [26]. Senescence-accelerated-prone mice 8 (SAMP8) and their controls, senescence-accelerated-resistant mice 1 (SAMR1) are good models for studying AD. SAMP8 have age-associated AD-like pathologic phenotypes, and exhibit deterioration in memory and learning [27, 28].

In this study, we evaluated the effects of GSE on human neuroblastoma SH-SY5Y cells, on *ex vivo* murine neurospheres, and on learning and memory in SAMP8 mice. Moreover, we performed immunohistochemistry in coronal brain sections to evaluate DG neurogenesis in SAMP8 mice. Finally, we assessed expression levels of genes associated with tumor necrosis factor-α (TNF-α), interleukin-6 (IL-6), dopamine and noradrenaline in murine brains.

## RESULTS

### The main component of GSE is anthocyanins

**Figure 1:**
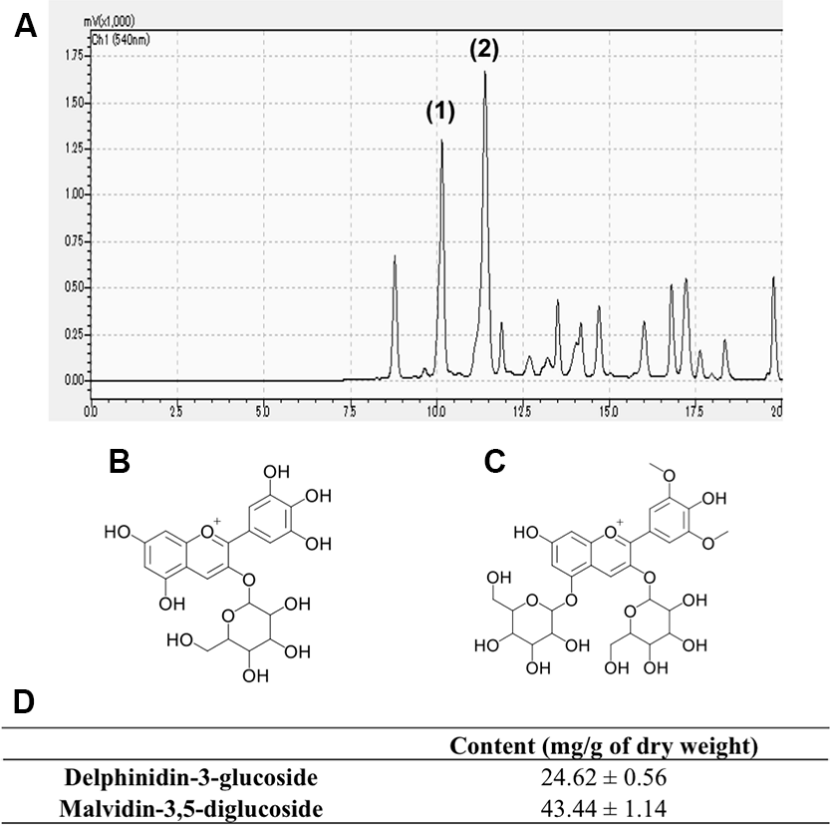
Representative chromatogram of Grape skin extract (GSE) (**A**). Anthocyanins content of GSE was determined by linear regression from the calibration graph of delphinidin-3-glucoside and malvidin-3,5-diglucoside. We determined that GSE contain (1) delphinidin-3-glucoside (**B**) and (2) malvidin-3,5-diglucoside (**C**). (**D**) Average contents of delphinidin-3-glucoside and malvidin-3,5-diglucoside in Grape skin extract (GSE).

Several anthocyanin peaks were successfully identified in GSE based on the retention time and spectral characteristics of their peaks against those of the standards using HPLC using a Shimadzu Prominence HPLC system. The range points of phenolic compound obtained by HPLC are presented in Figure 1A. Our result showed that the major anthocyanins presented in GSE were delphinidin-3-glucoside (Figure 1B) (24.66 mg/g of dry weight) and malvidin-3,5-diglucoside (Figure 1C) (43.4 mg/g of dry weight) as shown Figure 1D.

### Grape skin extract (GSE) and its major anthocyanins increased cell viability and protected SH-SY5Y cells against Aβ-induced cytotoxicity

**Figure 2:**
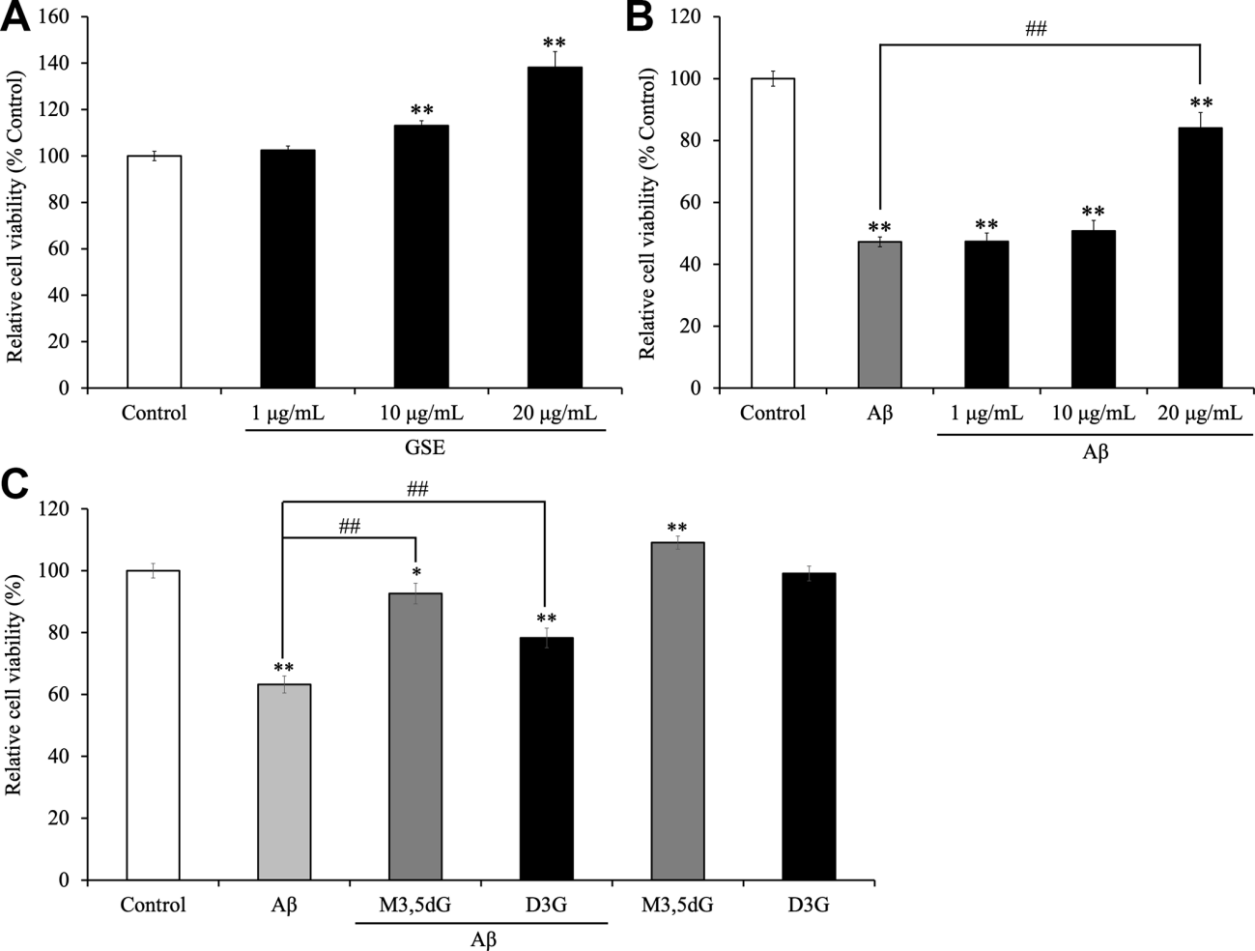
Effect of Grape skin extract (GSE) on (**A**) the cell viability and (**B**) amyloid-β_42_ (Aβ_42_)-induced changes in SH-SY5Y cells. And effect of malvidin-3,5-diglucoside (M3,5dG) and delphinidin-3-glucoside (D3G) on (**C**) the cell viability and amyloid-β_42_ (Aβ_42_)-induced cytotoxicity in SH-SY5Y cells. The cells were treated with or without 20 μg/mL GSE, 12.56 μM M3,5dG, 10.6 D3G, and 15 μM Aβ_42_ for 72 hr. Each bar represents the mean ± SEM (n = 5 independent experiments). ** P < 0.01 vs control cells, ## P < 0.01 vs Aβ_42_-treated cells.

First, to evaluate the cytotoxicity and mitochondrial activity of GSE, malvidin 3,5-diglucoside (M3,5dG) and delphinidin 3-glucoside (D3G) which are major anthocyanins in GSE, SH-SY5Y cells were treated with or without GSE (1, 10, and 20 μg/mL) or M3,5dG (12.56 μM) or D3G (10.6 μM) for 72 hr and then cell viability was measured using a MTT assay. One μg/mL GSE-treatment did not show effects on cell viability (Figure 2A). In contrast, 10 and 20 μg/mL GSE treated cells showed a significant increase in cell viability compared to the non-treated group (113.1 ± 2.1% and 138.2 ± 6.8%, respectively). Moreover, M3,5dG) treated cells also showed a significant increase (109.1 ± 2.1%) in cell viability compared with non-treated cells. However, delphinidin 3-glucoside (D3G) treated cells did not show any change of cell viability (99.1 ± 2.4%). Next, to evaluate the neuroprotective effect of GSE, SH-SY5Y cells were co-treated with GSE (20 μg/mL) and Aβ_42_ (15 μM) for 72 hr. The Aβ-treated group showed a significant reduction in cell viability compared to the non-treated group (47.3 ± 1.6%) but in contrast, co-treatment with 20 μg/mL GSE significantly ameliorated Aβ_42_-induced cytotoxicity (177.8% in Aβ_42_-treated cells) (Figure 2B). Also, we assessed the neuroprotective effect of malvidin 3,5-diglucoside (M3,5dG) and delphinidin 3-glucoside (D3G) on SH-SY5Y cells. Our results showed that M3,5dG (146.5%) or D3G (123.8%) significantly increase cell viability compared with Aβ_42_-treated cells (Figure 2C).

### Grape skin extract (GSE) increased intracellular ATP production and inhibited the increase intracellular ROS production induced by Aβ-treatment in SH-SY5Y cells

**Figure 3:**
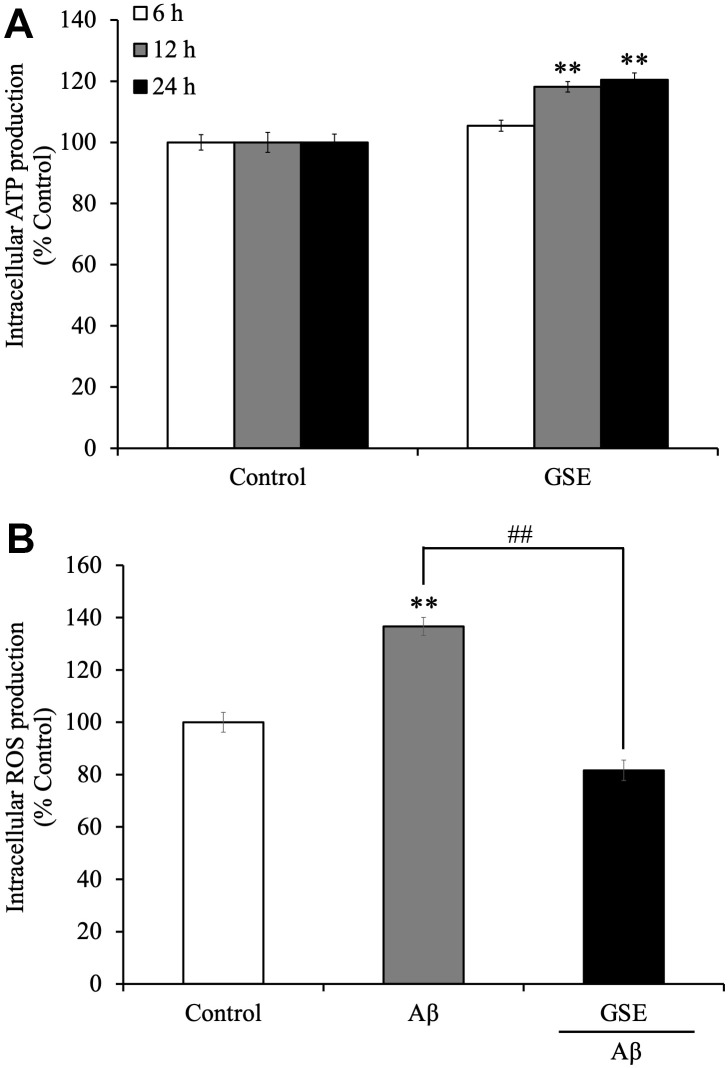
Effects of Grape skin extract (GSE) on (**A**) intracellular ATP and (**B**) reactive oxygen species (ROS) production of SH-SY5Y cells. The cells were treated with or without 20 μg/mL GSE for 6, 12, and 24 hr. After the treatment, intracellular ATP production levels were measured. And Intracellular ROS levels were detected by measuring fluorescence intensity of DCF oxidized by ROS. SH-SY5Y cells were pre-incubated with DCFH-DA for 1 h followed by treatment with growth medium or differentiation medium with or without 20 μg/mL GSE and 5 μM Aβ_42_ for 60 min. The fluorescence intensity was measured immediately after treatment. Data was set as % of non-treated cells (Control). Each bar represents the mean ± SEM (n = 5 independent experiments). ** P < 0.01 Compared with Control cells, ## P < 0.01 compared with Aβ_42_-treated cells by one-way ANOVA analysis.

Because we showed that GSE increased cell viability in SH-SY5Y cells, we considered whether GSE affects intracellular ATP levels. We found that 20 μg/mL GSE significantly increased intracellular ATP levels after 12 hr (118.2% compared to non-treated cells) and 24 hr incubation (120.4% compared to non-treated cells) (Figure 3A).

Moreover, we also tested the effect on intracellular ROS production in Aβ_42_-treated SH-SY5Y cells. Active oxidative phosphorylation in mitochondria produces ROS which have cytotoxic effects on neuronal cells. It was reported that Aβ increases oxidative stress, which induces cell death in neuronal cells [29]. Grape extract and anthocyanins have anti-oxidative activity [30], so there is a possibility that GSE can reduce oxidative stress induced by Aβ treatment. SH-SY5Y cells were treated with or without GSE or Aβ_42_ for 60 min. After 60 min of treatment, Aβ_42_-treated cells showed increased ROS production (136.6%) compared with non-treated cells (Figure 3B). However, 20 μg/mL GSE-treatment significantly decreased intracellular ROS production (59.8% compared to 100% in Aβ_42_-treated cells) (Figure 3B).

### Grape skin extract (GSE) increased neurosphere size

**Figure 4:**
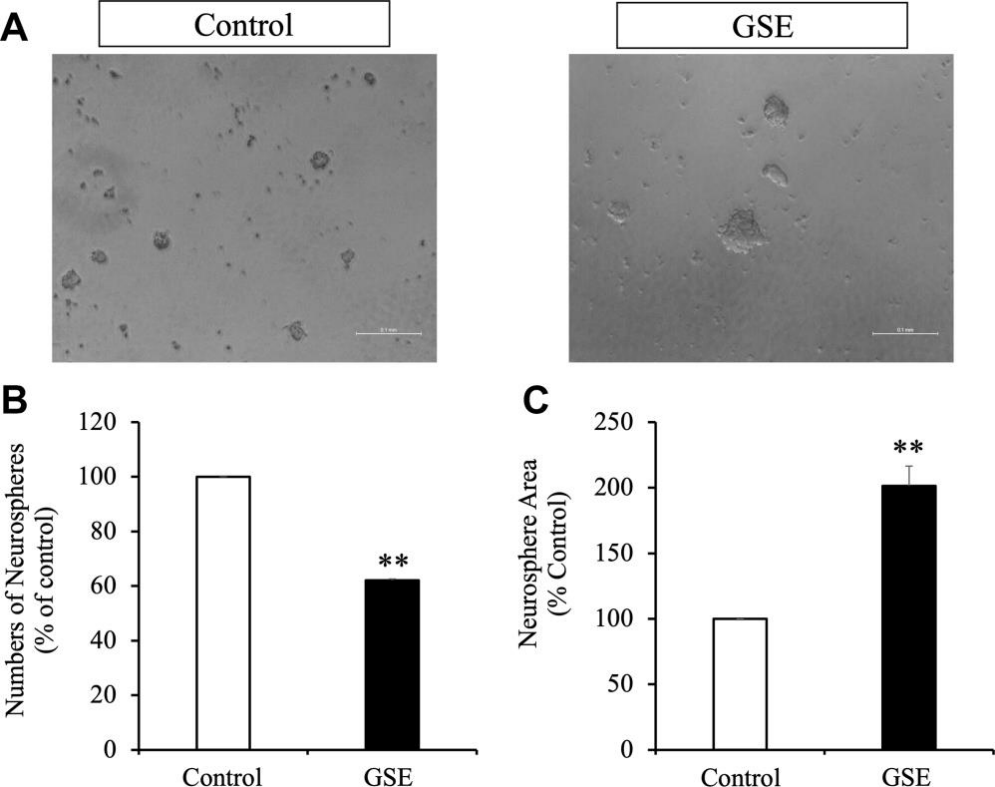
**Effect of Grape skin extract (GSE) on SVZ neurosphere proliferation.** Proliferation was tested using neurosphere cultures in presence of EGF and FGF2 (10 ng/mL). (**A**) Phase contrast microscopy images of neurospheres treated with or without grape extract (20 μg/mL) during 72 hr. Scale bar indicate 100 μm. (**B**) Neurosphere numbers after treatment with GSE. (**C**) The size of neurospheres after treatment with GSE. ** p < 0.01 compared with the control in a Student′s t-test.

To evaluate the effect of the GSE on NPCs proliferation, neurosphere assays were used. We measured the number and the size of newly formed neurospheres since the number of new neurospheres is an indicator of NPCs self-renewal capacity, and the size of neurospheres is an indicator of cell proliferation [31, 32]. We cultured NPCs during 72 hr in the presence or absence of the GSE (20 μg/mL) (Figure 4A). All experiments were performed in the presence of EGF (20 ng/mL) and FGF2 (10 ng/mL). GSE decreased the number of neurospheres generated (Figure 4B) but increased neurosphere size (Figure 4C).

### Administration of Grape skin extract (GSE) improved spatial learning and memory in SAMP8 mice

**Figure 5:**
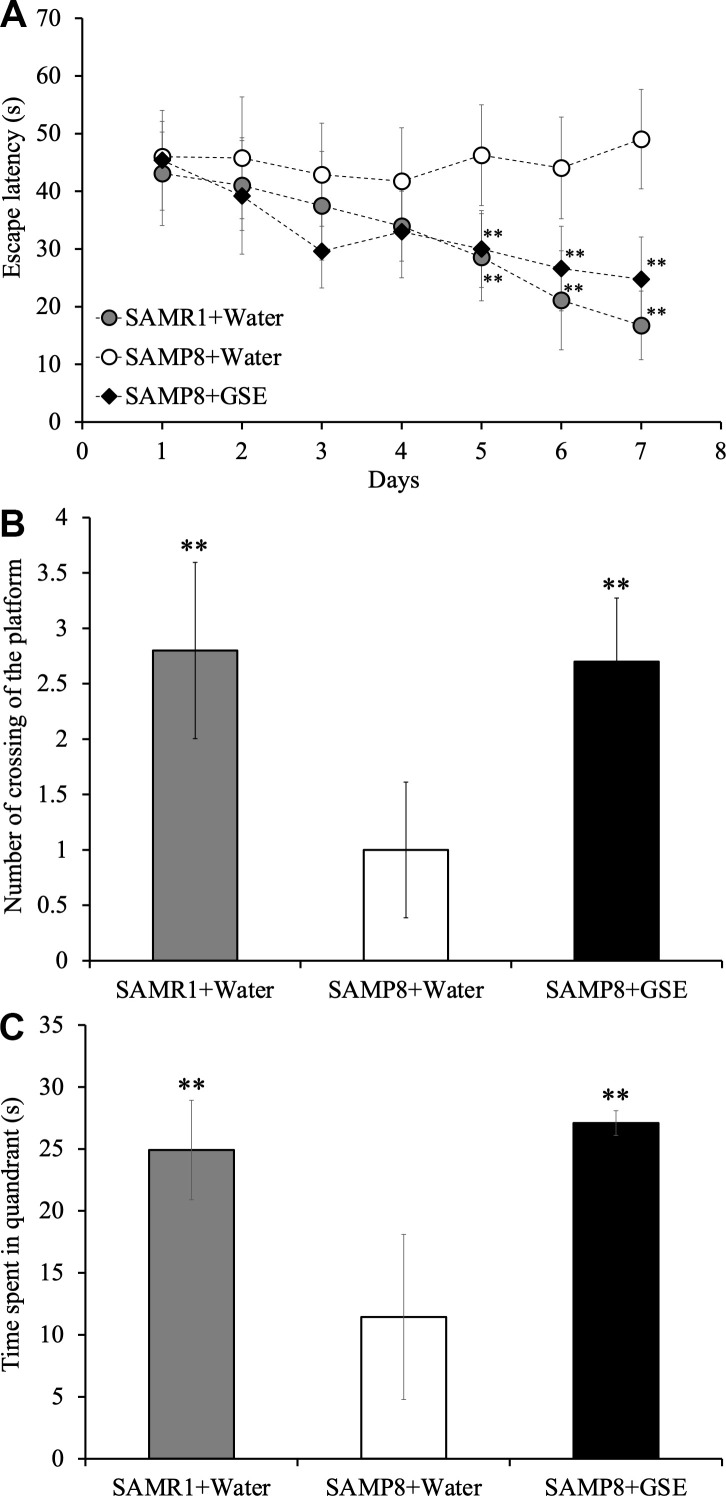
**Effect of Grape skin extract (GSE) administration on spatial learning and memory as evaluated by escape latency of senescence-accelerated resistant mouse 1 (SAMR1) mice, senescence-accelerated prone mouse 8 (SAMP8) mice, and SAMP8 50 mg/kg GSE-treated group by Morris water maze test.** (**A**) Effect of GSE on the time spent in the target quadrant. (**B**) Effect of GSE on numbers of crossings of platform by SAMR1 water-administered and SAMP8 GSE-treated or water-treated mice. (**C**) * P < 0.05, ** P < 0.01 Compared with SAMP8+Water group by one-way ANOVA analysis.

In order to evaluate the effect of GSE on spatial learning and memory impairment, which is a lineage-specific characteristic of SAMP8 mice, 50 mg/kg of GSE was orally administered to SAMP8 mice for 30 days. Then spatial learning and memory was evaluated by using the MWM test as reported [33]. Starting at day 5 and until day 7, both SAMR1 water-administered group (Day 5: 28.56 ± 7.55 s; Day 6: 21.10 ± 8.58 s; Day 7: 16.75 ± 5.95 s) and SAMP8 GSE-administered (50 mg/kg) group (Day 5: 30.00 ± 6.66 s; Day 6: 26.61 ± 7.33 s; Day 7: 24.75 ± 7.32 s) showed a statistically significant decrease in escape latency in comparison with SAMP8 water-administered group (Day 5: 46.25 ± 8.74 s; Day 6: 44.05 ± 8.81 s; Day 7: 49.05 ± 8.62 s) (Figure 5A).

Also, in order to verify whether the effect of spatial learning and memory improvement by GSE administration is sustainable and whether learning and memory ability of mice was dependent on spatial information, a probe test was carried out. In the probe test, the observation of the behavior of the mouse is performed under the same condition except removing the platform. As a result of probe test, Both SAMR1 water-administered group and SAMP8 GSE-administered group showed a significant increase both in the number of crossing times across the virtual platform (SAMR1 water-administered group: 2.8 ± 0.80; SAMP8 GSE-administered group: 2.7 ± 0.58) and the swimming time in the quadrant (SAMR1 water-administered group: 24.92 ± 4.01; SAMP8 GSE- administered group: 27.09 ± 1.00) where the platform was installed in comparison with SAMP8 water-administered group (number of crossing of the platform: 1.0 ± 0.61; time spent in quandrant: 11.44 ± 6.66 s) (Figure 5B, 5C).

### Grape skin extract (GSE) increased the number of mitotic cells in the DG

**Figure 6:**
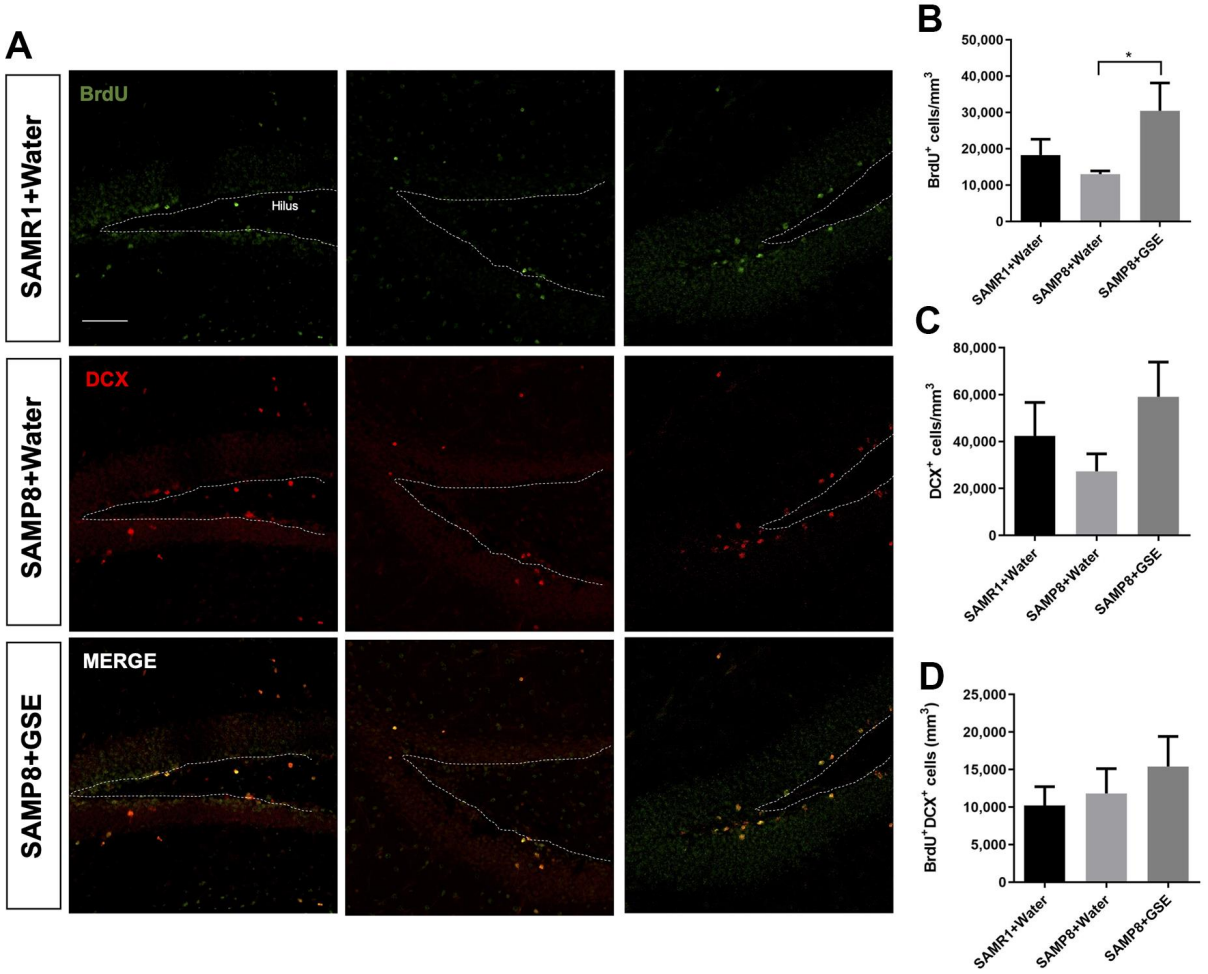
**Effect of oral administration of Grape skin extract (GSE) on dorsal dentate gyrus (DG).** SAMP8 mice were gavaged orally with GSE (50 mg/kg) for 30 days. (**A**) Photomicrograph shows adult mouse brain in coronal sections containing the DG processed for immunohistochemical detection of proliferating BrdU+ cells (green) and DCX (red). (**B**, **C**) Graphs represent the number of BrdU+ and DCX+ cells, respectively in the different treatment groups. (**D**) Graph represents the number of BrdU+ cells that co-express DCX in the different treatment groups. Each bar represents the mean ± SEM. * P < 0.05 compared with SAMP8+Water group by one-way ANOVA.

The neurosphere data and the GSE-induced improvement of learning and memory suggested that *in vivo* proliferation might be increased in the DG stem cell niche by GSE (Figure 6). To test this, we used the same mice as for the MWM which were administered GSE (gavage) for 30 days. BrdU, an analog of thymidine, is incorporated into cells in active S phase of the cell cycle, and was used to evaluate proliferation in the neurogenic niches. BrdU was provided to the animals during 9 consecutive days starting the 14th day of treatment with GSE, diluted in drinking water. We also studied the effect of the GSE on BrdU+ cells that co-express the young neuron marker doublecortin (DCX). In the GSE-administered SAMP8 group (n = 5), we found a significant increase in the number of BrdU+ cells compared to the SAMP8 water administered group (n = 5) (Figure 6B). We also observed a trend for increased number of neuroblasts (DCX+) in GSE administered SAMP8 mice compared with both SAMR1 and SAMP8 water administered groups (Figure 6C). However, this increase was not statistically significant. There was also a non-statistically significant trend for more newborn neurons (BrdU+DCX+) cells in the GSE treated SAMP8 mice compared to the other two groups (Figure 6D).

### GSE suppressed gene expression and protein levels of neuroinflammatory factors in the brain of SAMP8 mice

**Figure 7:**
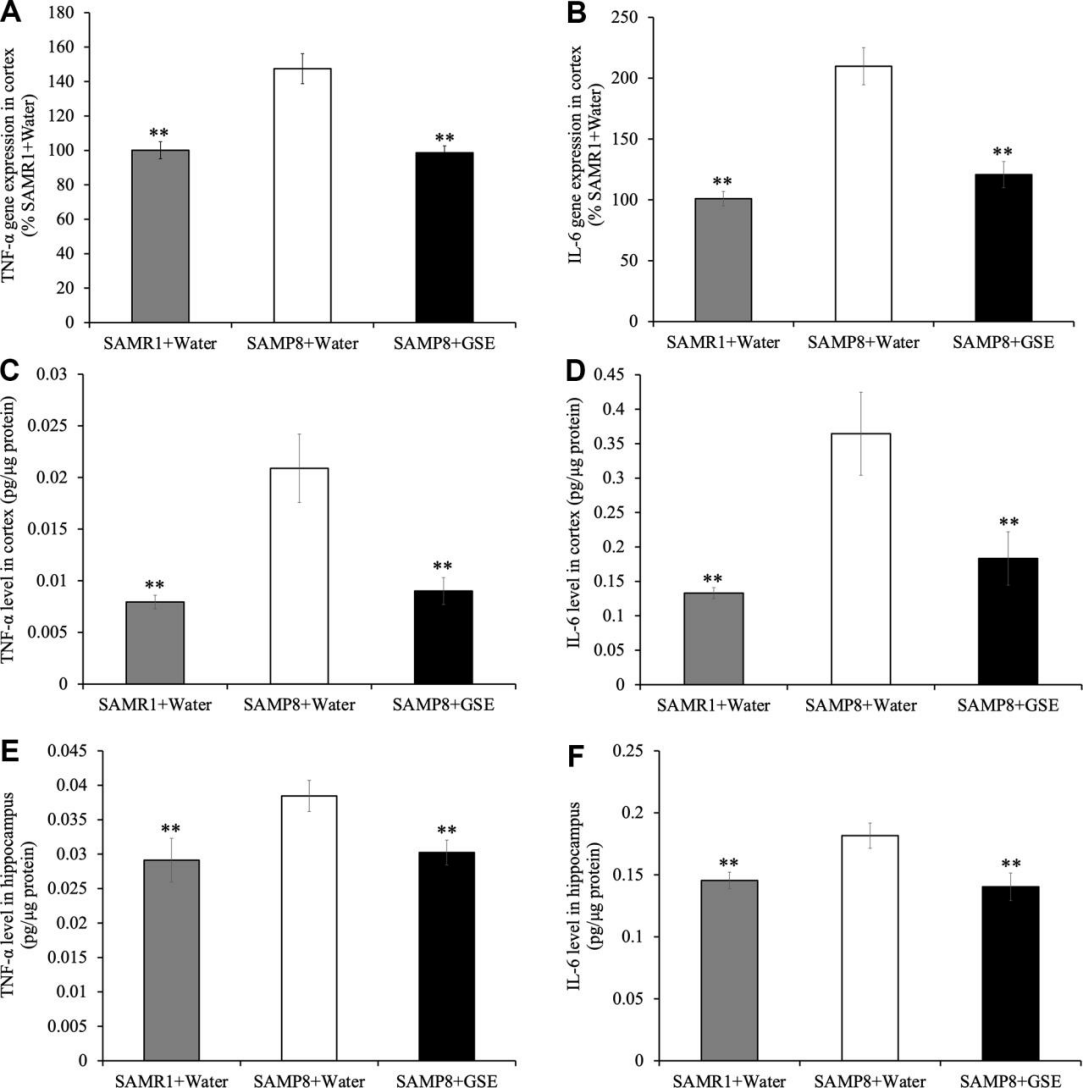
Effect of Grape skin extract (GSE) on gene expression of (**A**) tumor necrosis factor-α (TNF-α) and (**B**) interleukin-6 (IL-6) and protein levels of TNF-α and IL-6 in the cerebral cortex (**C**, **D**) and the hippocampus (**E**, **F**) of SAMP8. SAMP8 mice were orally administered with 50 mg/kg GSE for 30 days. The gene expression of TNF-α and IL-6 were evaluated using real-time RT-PCR. And protein levels of TNF-α and IL-6 were determined by ELISA kit. Each value is presented as mean ± SD. ** P < 0.01 compared to SAMP8+Water by one-way ANOVA.

To investigate if altered neuroinflammation may have contributed to the spatial learning and memory improvement of GSE, gene expression analysis of inflammatory cytokines in the cerebral cortex was performed. Analysis using the real-time RT-PCR revealed that gene expression levels of TNF-α and IL-6, which are major inflammatory cytokines, were greater in the SAMP8 water-administered group by 147.46% and 209.81% respectively as compared with the SAMR1 water-administered groups (Figure 7A, 7B). However, in the cerebral cortex of the SAMP8 GSE-administered group, a decrease in gene expression levels of TNF-α (66.89% compared to 100% in SAMP8 water-administered group) was observed (Figure 7A). Also, GSE administration significantly decreased IL-6 gene expression (57.56% compared to 100% in SAMP8 water-administered group) (Figure 7B).

Moreover, protein levels of TNF-α and IL-6 of the cerebral cortex (TNF-α: 0.021 ± 0.00066 pg/μg protein, IL-6: 0.18 ± 0.030 pg/μg protein) and the hippocampus (TNF-α: 0.038 ± 0.0023 pg/μg protein, IL-6: 0.18 ± 0.010 pg/μg protein) in SAMP8 water-administered group were also significantly increased compared with the SAMR1 water-administered groups in the cortex (TNF-α: 0.0080 ± 0.00066 pg/μg protein, IL-6: 0.067 ± 0.0040 pg/μg protein respectively) and hippocampus (TNF-α: 0.029 ± 0.0032 pg/μg protein, IL-6: 0.15 ± 0.0068 pg/μg protein respectively) (Figure 7C–7F). However, GSE-administration decreased the levels of TNF-α and IL-6 in the cerebral cortex (TNF-α: 0.0045 ± 0.00065 pg/μg protein, IL-6: 0.092 ± 0.019 pg/μg protein respectively) and the hippocampus (TNF-α: 0.030 ± 0.0018 pg/μg protein, IL-6: 0.14 ± 0.011 pg/μg protein respectively) compared with SAMP8 water-administered group (Figure 7C, 7D).

### GSE increased neurotransmitter concentration in the cerebral cortex of SAMP8 mice

**Figure 8:**
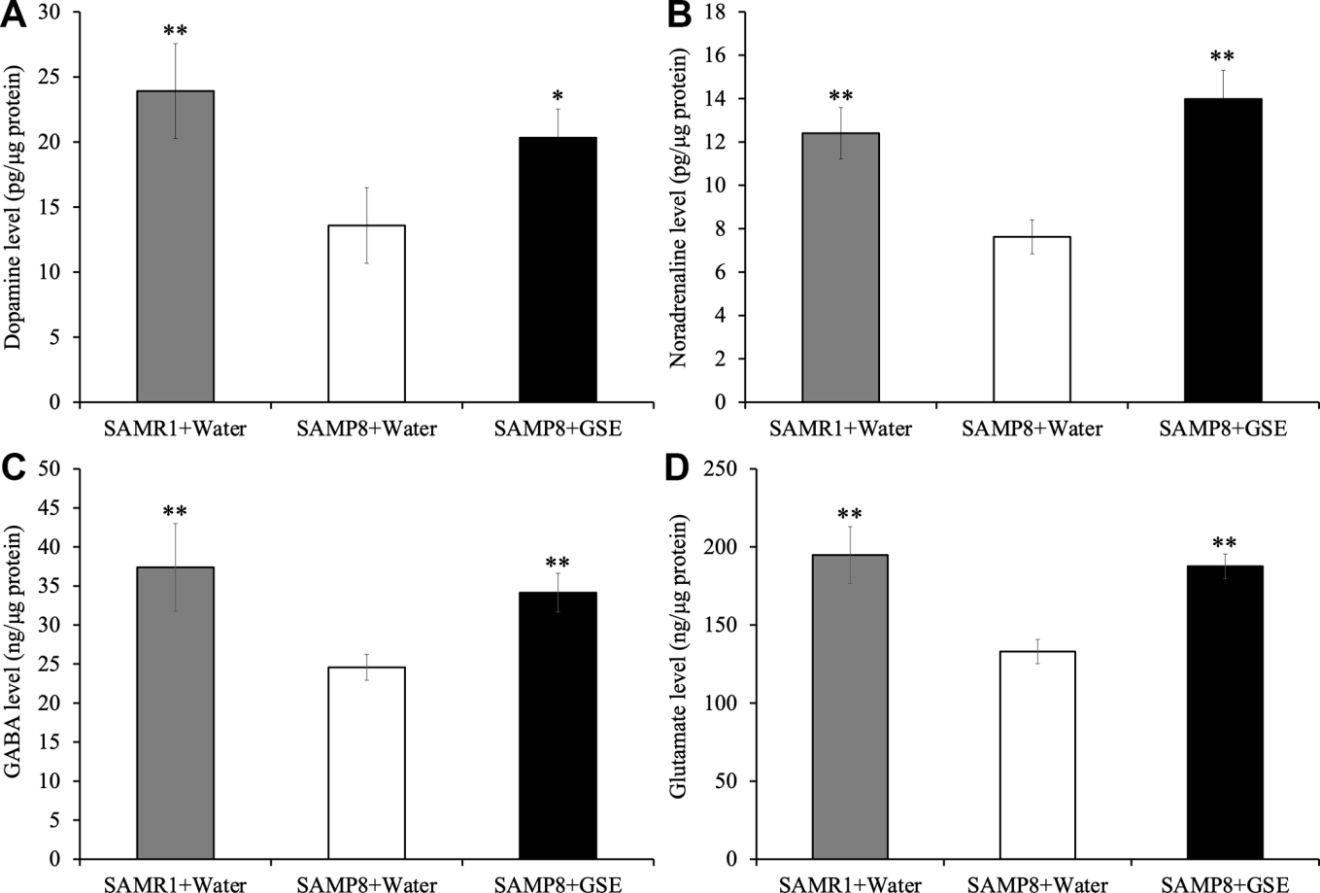
Effect of Grape skin extract (GSE) on (**A**) dopamine, (**B**) noradrenaline, (**C**) GABA, and (**D**) glutamate levels in the cerebral cortex of SAMP8. SAMP8 mice were orally administered with 50 mg/kg GSE for 30 days. These neurotransmitters levels were determined by ELISA kit. Each value is presented as mean ± SD. ** P < 0.01 compared with SAMP8+Water by one-way ANOVA.

To further probe potential mechanisms contributing to the spatial learning and memory improvement of GSE, we quantified neurotransmitters in the cerebral cortex of SAMP8 and SAMR1 mice. Using ELISAs, we found that the levels of dopamine (13.58 ± 2.90 ng/μg protein), noradrenaline (7.62 ± 0.78 ng/μg protein), GABA (24.57 ± 1.65 ng/μg protein), and glutamate (132 ± 7.76 ng/μg protein) in the cerebral cortex were significantly decreased in the SAMP8 water-administered group compared with the SAMR1 water-administered group (23.91 ± 3.64 ng/μg protein, 12.4 ± 1.19 ng/μg protein, 37.39 ± 5.6 ng/μg protein, and 194.8 ± 18.21 ng/μg protein respectively) (Figure 8A). However, in the cerebral cortex of the SAMP8 GSE-administered group, dopamine (20.33 ± 2.2 ng/μg protein), noradrenaline (13.98 ± 1.32 ng/μg protein), GABA (34.14 ± 2.48 ng/μg protein), and glutamate (187.62 ± 7.85 ng/μg protein) levels were significantly increased compared with the SAMP8 water-administered group (Figure 8).

## DISCUSSION

In this study, we focus on Grape skin extract (GSE) which is rich in anthocyanin, one of the flavonoids. We evaluated GSE’s neuroprotective effect on human neural SH-SY5Y cells and stem cell properties in murine neurosphere primary cultures. Moreover, we evaluated the effect of GSE on learning and memory and *in vivo* neurogenesis in SAMP8 mice, a good model for aging related brain dysfunction. SAMP8 is also a good model of AD, and thus we focused on major pro-inflammatory cytokines (TNF-α and IL-6) and neurotransmitters (dopamine and noradrenaline), which are affected by Aβ toxicity.

Neuroprotection is a widely studied treatment option for central nervous system (CNS) disorders including AD. Neuroprotection aims to prevent or delay disease progression and loss of neurons. Common mechanisms behind neurodegeneration include oxidative stress, mitochondrial dysfunction, excitotoxicity, inflammation, and abnormal protein aggregation [34, 35]. Among these mechanisms, neuroprotective therapy often targets oxidative stress, an important cause of CNS disorders. Limiting oxidative stress is important for protection against neuronal death. In the present study, GSE-treated SH-SY5Y cells exhibited reduced Aβ-induced neurotoxicity - a neuroprotective effect. Moreover, malvidin 3,5-diglucoside (M3,5dG) and delphinidin 3-glucoside (D3G), major components of GSE, showed neuroprotection against Aβ-induced neurotoxicity. Interestingly, GSE also increased cell SH-SY5Y viability compared with non-treated cells. Since the MTT assay reflects mitochondrial reductase activity, this result indicated that GSE may activate mitochondrial function. Actually, our ATP assay showed that GSE treatment significantly increases intracellular ATP production. Therefore, GSE may have the potential to enhance energy metabolism in neuronal cells.

Brain aging is characterized by progressive metabolic imbalances, alterations in cerebral vasculature, and decreased adult neurogenesis including the decline of the numbers and functions of NSCs and NPCs [36, 37]. All of these changes may contribute to cognitive decline, impaired learning, loss of working and transient memory and motor coordination [38]. Neurogenesis occurs throughout the life of mammals, in the embryonic, fetal, neonatal, adult and aging periods. A recent report showed decline in neurogenesis markers with age, especially in AD [37]. In this study, we isolated NSCs and NPCs from the mouse brain and grew neurospheres as an *in vitro* neurogenesis model using the growth factors EGF and FGF2 to promote proliferation and neurosphere formation. Our result showed that the GSE-treatment significantly decreased the number of neurospheres, but increased their size. This result suggested decreased stem cell self-renewal but increased NPCs proliferation, respectively. Increased neurosphere size is a common, but indirect, read-out of proliferation and future work will confirm whether *in vitro* proliferation is altered in neurospheres.

The SAMP8 mouse is accelerated senescence strain spontaneously developed from breeding pairs of AKR/J series mice [39]. The SAMP8 mouse develops early learning and memory impairments (8 to 10 months) with other characteristics similar to Alzheimer's disease [40]. SAMP8 mice accumulate Aβ in the brain, where it forms senile plaques [41]. Other SAMP8 AD characteristics include hyperphosphorylation of Tau [42], oxidative damage [43], decreased choline acetyl transferase activity [44], increased glutamate [45], altered NMDA receptor function [46] and increased neuronal nitric oxide synthase [47]. Therefore, SAMP8 mice are considered a good animal model for AD neuropathology and functional loss. Our MWM test results showed that 30 days of GSE improves spatial learning memory of SAMP8 mice. Also, probe test results showed that spatial learning and memory is improved by GSE is retained. We examined the effect of GSE *in vivo* on adult neurogenesis with immunohistochemical analysis of the DG neurogenic stem cell niche in SAMP8 mice. We observed a significant increase in BrdU+ cell numbers in GSE-administered SAMP8 mice. When we prepared the BrdU in water, 1% sucrose was added to induce drinking similar to our previous publications and we consistently observe similar numbers of BrdU+ cells within groups suggesting that drinking behavior and therefore BrdU dose was not altered [33, 48, 49]. It is also formally possible that GSE increased drinking of water, leading to more BrdU+ cells, however there is no a priori reason to believe this was the case. There was also a trend for increased numbers of DCX+ and BrdU+DCX+ DG cells in SAMP8 mice after GSE treatment. A larger sample size may show GSE significantly increases DCX+ cell numbers. Future studies may also show that higher doses of GSE significantly increase hippocampal neurogenesis. However, based on this study, the data suggest that the improvement in memory may not be due to augmented hippocampal neurogenesis.

SAMP8 mice develop symptoms similar to AD, including cognitive impairment due to oxidative stress damage, neuroinflammation due to increased inflammatory cytokines, and reduced neurogenesis in older mice [50, 51]. Neuroinflammation and overactivation of microglia are closely interrelated, and the latter is widely accepted as a major hallmark of CNS ROS generation (CNS) [52, 53]. Intercellular ROS generation leads to the release of inflammatory mediators via MAPKs and NF-κB signaling molecules in microglia [54]. Microglia-derived elevation of free radical generation damages neuronal cells, and is implicated in neurodegeneration [55]. Also, excessive production of inflammatory cytokines downstream of MAPK and NF-κB induces mitochondrial dysfunction and damage to dopaminergic neurons [56]. Oxidative damage is enhanced in SAMP8 brains and inflammatory TNF-α and IL-6 increases in the hippocampus and cortex [57]. In the present study, we confirmed that the SAMP8 water-treated group had significantly increased gene expression and protein levels of TNF-α and IL-6 in the cerebral cortex and the hippocampus. SAMP8 mice have a neuropathological phenotype described above suggesting inflammatory cytokines may be expressed at higher levels in the neurogenic niches. Therefore, because inflammation, TNF-α and IL-6 decrease neurogenesis [58], GSE treatment could affect neurogenesis via these molecules [59, 60]. In future studies, we will evaluate levels of anti-inflammatory cytokines and predict these are augmented by GSE. GSE and its major compounds, M3,5dG and D3G, also showed neuroprotective effects through the suppression of Aβ-induced ROS production *in vitro* in SH-SY5Y cells. Moreover, GSE-administration rescued the decreases in dopamine, noradrenaline, GABA and glutamate levels of SAMP8 mice. Dopamine and noradrenaline are synthesized by specific neurons and play important roles in mood, cognitive and motor function. Impairment of the dopaminergic system may cause depression [61], memory loss [62] and impaired motor control in AD. Glutamate is the most abundant excitatory CNS neurotransmitter and is the principal neurotransmitter of cortical efferents. GABA, the main inhibitory CNS neurotransmitter, and defective balance of glutamate and GABA contributes to AD symptoms [63]. Thus, our neurotransmitter results indicated that GSE may improve neurotransmitter impairment in SAMP8 mice. Taken together, our results suggested that GSE may have anti-oxidative effects *in vivo* and decreased oxidative stress associated with inflammatory cytokines in aging thus protecting neurons through suppressing neuroinflammation.

Our chemical analysis using HPLC, confirmed that GSE contains large proportions of malvidin-3,5-diglucoside and delphinidin-3-glucoside. Delphinidin and malvidin are major anthocyanin components of red wine and berries and both are amongst the richest anthocyanins found in most plants [64]. In the present study, our results showed that both M3,5dG- and D3G-treatment have neuroprotective effects on Aβ_42_-treated SH-SY5Y cells. Based on the present study, it was predicted that M3,5dG and D3G, which are derived from GSE, have a neurophysiological activation effect. Several bioavailability studies showed that anthocyanins, such as malvidin-3-glucoside and D3G, are absorbed in plasma and are able to reach the brain in unchanged forms [65–68]. Interestingly, other reports indicated that several anthocyanins (malvidin-3-glucoside, delphinidin-3-galactoside, etc.) can reach the cerebellum, cortex, hippocampus and striatum region of brain of rat [66]. However, there are no bioavailability studies specifically showing that M3,5dG can reach the brain or not. Therefore, in future research, further studies on brain function and protection from pathology of anthocyanins will be necessary.

These findings suggest that GSE has an effect on proliferation, protection of neurons from oxidative stress and neuroinflammation associated with aging, and increases learning and memory and brain neurotransmitters. It could be used as a new therapeutic agent for the treatment and prevention of neurodegenerative diseases related to the ageing process. It is thus crucial to continue the study and discovery of the effects of GSE and its active compounds un other aspects of neural health and pathology.

## MATERIALS AND METHODS

### Preparation of Grape skin extract (GSE), malvidin 3,5-diglucoside (M3,5dG), and delphinidin 3-glucoside (D3G)

The grape skins used in this study, were from Japanese grape varieties and obtained from ZEON CORPORATION (a hybrid between Vitis flexuosa and Vitis vinifera ‘Merlot’ by SHIMURA Grape Laboratory). Extraction from the grape skin was carried out by homogenizing 1 g with 10 mL volume 1% formic acid/99% MeOH, then subjected to an ethyl acetate liquid/liquid extraction method [69] to obtain the anthocyanin-rich fraction (GSE). The anthocyanin-rich fraction was concentrated using vacuo and freeze dried. For *in vitro* experiments, 100 mg of the freeze dried GSE was dissolved in 1 mL volume of 70% ethanol. and for *in vivo* experiment, 15 mg of the freeze dried GSE was dissolved in 10 mL volume of milliQ water with sonication.

Malvidin 3,5-diglucoside (M3,5dG) and Delphinidin 3-glucoside (D3G) were purchased from FUJIFILM Wako Pure Chemical Corporation (Osaka, Japan). Both compounds were dissolved in 70% ethanol.

### Analysis of Grape skin extract (GSE) using high performance liquid chromatography (HPLC)

GSE was analyzed by HPLC using a Shimadzu Prominence HPLC system (Shimadzu Corp., Kyoto) equipped with a quaternary solvent delivery system, an auto-sampler, and a DAD detector. Samples were resolved on a ZORBAX SB-C18 column (4.6 mm I.D. x 250 mm x 3.5 μm particle size) (Agilent Technologies Inc, CA, USA). The mobile phase (A : 5% formic acid, B : ACN/MeOH, 1:1 ratio, v/v) consisted of a 0 - 100% gradient of A for 0 - 40 min. Chromatography was carried out in gradient mode, using a flow rate of 1.0 ml/min at 30° C. The wavelength was set at 540 nm for monitoring anthocyanins. Five concentrations of each anthocyanin standards (Malvidine-3,5-diglucoside, and Delphinidine-3-glucoside) were prepared, with 10 μl of each injected as external standards. GSE references to the anthocyanin standard calibration curve were analyzed in triplicate.

### SH-SY5Y cell culture

The human neuroblastoma SH-SY5Y cell line was purchased from American Type Culture Collection. SH-SY5Y cells were cultured in a 1:1 (v/v) mixture of Dulbecco’s modified Eagle Medium and Ham’s F-12 medium (Gibco, Japan) supplemented with 15% heat-inactivated fetal bovine serum (Bio West, U.S.A) and 1% penicillin (5000 μg/ml)–streptomycin (5000 IU/ml) (PS) (Lonza, Japan) at 37° C in a humidified atmosphere of 5% CO_2_ in air. SH-SY5Y cells were cultured in 100-mm petri dishes or 96-well plates. Serum-free Eagle’s minimum essential medium (OPTI-MEM; Gibco, Japan) was used to culture the cells for the cell viability assay.

### MTT assay for neuroprotection

Cell viability and mitochondrial activity were determined using a 3-(4,5-dimethylthiazol-2-yl)-2,5-diphenyltetrazolium bromide (MTT) assay to check for effects of GSE, M3,5dG, D3G, and Aβ (15 μM) on cytotoxicity. Cell viability was measured using the MTT method. SH-SY5Y cells were seeded at 2×10^5^ cells/mL in 96-well plate (BD BioCoat, USA) and incubated for 24 hr. After 24 hr incubation, SH-SY5Y cells were treated with GSE or 15 μM Aβ (FUJIFILM Wako Pure Chemical Corporation, Japan) for 72 hr. To evaluate the neuroprotective effects of GSE against Aβ-induced cytotoxicity, SH-SY5Y cells were treated with or without 1, 10, and 20 μg/mL GSE, or 12.56 μM M3,5dG or 10.6 μM D3G, and 15 μM Aβ for 72 hr. The concentration of M3,5DG and D3G were determined from the result of chemical analysis. Control cells were treated with the same volume of 70% EtOH as the GSE-treated cells (final concentration: 0.07%). After sample treatment, a solution of 5 mg/ml MTT dissolved in PBS was added (10 μl/well) and incubated for another 24 hr. The resulting MTT formazan was dissolved in 100 μl of 10% SDS (w/v) and the absorbance was measured using a microtiter plate reader (Dainippon Sumitomo Pharma Co., Ltd., Japan).

### ATP assay

The effect on ATP production of GSE in SH-SY5Y cells was determined using a luciferase luminescence assay kit (ATP reagents for cell: TOYO Ink, Tokyo, Japan). SH-SY5Y cells were seeded at 2×10^5^ cells/mL and incubated for 24 hr. After incubation, SH-SY5Y cells were treated with Opti-MEM with or without 20 μg/mL GSE for 6, 12, 0r 24 hr. Control cells were treated with the same volume of 70% EtOH as the GSE-treated cells (final concentration: 0.07%). After 6, 12, or 24 hr incubation, ATP assay reagent was added (100 μl/well) and incubated for 10 min at room temperature while avoiding light exposure. After incubation, the solution was transferred into a white clear-bottom 96-well plate (BD Falcon) and luminescence was detected using a microplate reader.

### Reactive oxygen species (ROS) assay

The effect of GSE on intracellular ROS levels was determined using the OxiSelect Intracellular ROS assay kit (Cosmo Bio, Japan) following the previous study [70]. SH-SY5Y cells were seeded at 2.0×10^5^ cells/mL in black clear-bottom 96-well plates. After 24 hr incubation, cells were pre-treated with 100 μl/well 1X DCFH-DA (1 mM DCFH-DA in medium) and incubated for 1 hr avoiding light exposure. After the incubation, cells were washed twice with PBS and treated with Opti-MEM with or without 20 μg/mL GSE or 5 μM Aβ and incubated for 60 min avoiding light exposure. Control cells were treated with the same volume of 70% EtOH as the GSE-treated cells (final concentration: 0.07%). After treatment, fluorescence intensity (480 nm/530 nm) was measured using a microplate reader. The fluorescence intensity was calculated as a percentage of the non-treated cells.

### Neurosphere formation assay

NSCs and NPCs were obtained from postnatal (P7) mice SVZ following the procedure described by Doetsch et al. [71, 72] and modified by Torroglosa et al. [32]. Six animals were used for each independent culture. Cultures of neurospheres were maintained in defined medium (DM) composed of Dulbecco′s modified Eagle′s medium/F12 medium (1:1 v/v) with 1 mg/L gentamicin (GIBCO) and B27 supplement (Invitrogen, Carlsbad, CA, USA). EGF (20 ng/mL, GIBCO) and FGF2 (10 ng/mL, Peprotech, Frankfurt, Germany) were added to cultures for culture expansion.

To evaluate the effect of GSE on cell proliferation, single cells from mechanically disaggregated primary neurospheres were seeded in anti-adherent 96-well plates (Corning, NY, USA) at a density of 20,000 cells/mL. GSE (20 μg/mL) was added at the time of seeding and also, EGF (20 ng/mL, GIBCO) and FGF2 (10 ng/mL, Peprotech, Frankfurt, Germany). 72 hr after seeding, the number and size of neurospheres was measured using a phase contrast microscope and ImageJ software. Each condition was performed in technical triplicate and repeated at least three independent times.

### Animals and sample treatment

Male SAMP8 mice, aged 16 weeks, were obtained from Japan SLC, Inc. (Shizuoka, Japan) and housed under conditions of controlled temperature and humidity and unrestricted access to food and water with a 12-hr light/dark cycle. Senescence-accelerated mouse resistant 1 (SAMR1), SAM lineage, were used as a normal aging control, were housed the same as SAMP8 mice. The protocol for this animal experiment was approved by the Animal Care and Use Committee of the University of Tsukuba (17-062). After 1 week of acclimatization to the laboratory conditions, the mice were divided into three groups: SAMR1 water-administered group (n = 10), SAMP8 water-administered group (n = 10), and SAMP8 GSE-administered group (n = 10). The GSE-administered group were administered 50 mg/kg GSE mixed with MilliQ water for 30 days by an oral gavage using tube and syringe. In previous animal experiments on physiological activities of grape extract, doses around 50 mg/kg were effective [73–75]. Considering these previous studies, we set the concentration of GSE to 50 mg/kg. Water-administered groups were administered an equivalent volume of MilliQ water. In addition, BrdU (1 mg/ml) was diluted in the drinking water and provided to the SAMR1-water administered group, the SAMP8-water administered group, and the GSE-administered group during 9 consecutive days starting with the 14th day of oral administration.

### Morris water maze (MWM) test

**Figure 9:**
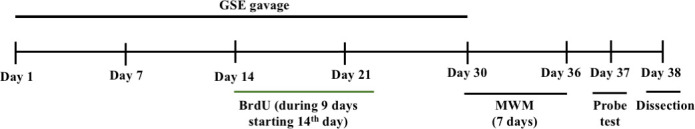
The experimental design of our *in vivo* experiment.

To evaluate the effects on spatial learning and memory of GSE administration, the MWM test was performed as previously described [33]. The apparatus consisted of a circular water tank (120 cm in diameter and 45 cm in height) that contained water (23 ± 2° C) to a depth of 30 cm and divided into four quadrants designated as north, east, west, and south. The escape platform (10 cm in diameter) was submerged 1 cm below the water surface and placed at the midpoint of any quadrant so that it was invisible at water level. All mice received training for 7 days (30 – 36th day) (Figure 9) consecutively using a single hidden platform in one quadrant, with the start point rotating round the other three quadrants. The latency to escape from the water maze (find the hidden escape platform) was measured for each trial. Then, a probe test was performed after 24 hr on the 37th day to test memory consolidation (Figure 9). At the probe test at day eight after MWM test training session, the platform was removed and each mouse were allowed to swim freely for 60 s. The respective time spent by each mouse in the target quadrant and the number of crossings over the platform location (where the platform was located during the training) was calculated. The time spent by each mouse in the target quadrant and number of times crossing the virtual platform are considered to represent the degree of memory consolidation.

### Tissue processing and immunohistochemistry

Animals were sacrificed by cervical dislocation, brains removed and fixed in 4% PFA for 2 weeks and then cryoprotected in 30% sucrose (w/v) in PBS for 48 hr, both at 4° C. Using a microtome, serial 30 μm coronal brain sections were obtained. Sections were stored at -20° C in cryoprotectant solution (ethylene glycol, glycerol, 0.1 M phosphate buffer, pH 7.4, 1:1:1:2 by volume). For immunohistological detection of BrdU, antigen retrieval was achieved with 1 M HCl at 37° C for 1 h. After washing in PBS, sections were blocked with a solution composed with PBS, 0.1% Triton X-100 and 1% bovine serum albumin (Sigma) for 1 hour. Primary antibodies were incubated overnight at 4° C. Sections were washed and incubated with fluorochrome-conjugated specific secondary antibodies overnight at 4° C. Slices were put on slides and mounted in Prolong Antifade Kit (Molecular Probes, Eugene, OR, USA). Confocal images were obtained with a Zeiss LSM 710 laser scanning confocal microscope using the Z-stack and tile functions as appropriate. Primary antibodies used were sheep polyclonal anti-BrdU (1:500, Abcam) and goat anti-doublecortin (DCX, 1:200, Santa Cruz, CA, USA).

### Quantitative analyses and stereology

For cell counting stereological methods were used to estimate the number of positive cells to each specific marker analyzed following the procedure described by Geribaldi-Doldán et al., 2018 [76]. 5 animals were used per condition in order to evaluate adult neurogenesis. SGZ cells were counted in one out of every five 30 μm thick-serial coronal sections. In each section, cells were counted in the SGZ of the DG. At least 14-16 sections per brain were analyzed under confocal microscopy at 40x magnification. The total number of cells were referred to the volume analyzed. Confocal images of each section were obtained using a Zeiss LSM 719 microscope using the Z-stack function. Mouse treatment was coded depending and quantification was done in a blinded analysis.

### RNA extraction from the cerebral cortex of mouse brain

Also, to elucidate the mechanism of spatial learning and memory improvement effect of GSE administration, animals were sacrificed and the brain was carefully removed after the behavioral test. The cerebral cortex was carefully dissected, frozen in liquid nitrogen and stored at -80° C until use. 100 mg of the cerebral cortex was homogenized in 1 ml of ISOGEN Reagent (NipponGene, Tokyo, Japan) using a glass-teflon homogenizer. RNA extraction was performed according to the protocol of the ISOGEN Kit (NipponGene). Briefly, 0.2 ml of chloroform (Wako, Japan) was added to the homogenized sample and centrifuged (12000 × g, 15 min, 4° C.) to isolate and collect the aqueous phase, then 0.5 mL of isopropanol (Sigma) was added to elute the RNA. After washing with 70% ethanol, the RNA solution was dissolved in Tris-EDTA buffer solution pH 8.0 (Sigma) was quantified by using a Nanodrop 2000 spectrophotometer (Thermo Fisher Scientific, Waltham, MA, USA).

### TaqMan quantitative RT-PCR analysis of gene expression in the cerebral cortex of mouse brain

The RNAs were then used as templates for reverse transcription polymerase chain reaction (RT-PCR) using the Superscript III reverse transcriptase kit (Invitrogen, USA) following the manufacturer’s instructions. Specific TaqMan probes for tumor necrosis factor-α (TNF-α) (Mm00447557_m1) and Interleukin-6 (IL-6) (Mm00500992_m1) (Applied Biosystems, USA) were used and real-time polymerase chain reaction (rt-PCR) analysis was performed with a 7500 Fast Real-Time PCR system using TaqMan Universal PCR mix at the following thermal cycling protocol: 95° C for 10 min followed by 40 cycles of 95° C for 15 s and 60° C for 1 min. The Actin beta was used as the internal control. Data were analyzed using 7500 Fast System SDS Software 1.3.1. (Applied Biosystems).

### TNF-α and IL-6 analysis using ELISAs

Commercial ELISA kits (R&D systems, USA) were used to measure TNF-α and IL-6 levels in the cerebral cortex of mice brains. Briefly, cerebral cortex and hippocampus were isolated from brain and homogenized in RIPA buffer with protease inhibitor (Santa Cruz Biotechnology, Japan). After centrifugation (10,000 x g, 30 min), 50 μL supernatant from cortex (protein samples) or 100 μL supernatant from hippocampus or 100 μL standards were used according to the manufacturer’s instructions. TNF-α and IL-6 levels were computed by correcting for the protein concentration. Protein estimation was conducted using 2-D quant kit (GE Healthcare Inc., Japan) according to procedure of the kit and the data were expressed as pg/μg protein.

### Dopamine, Noradrenaline, gamma aminobutyric acid (GABA), and Glutamate analysis using ELISAs

Dopamine, noradrenaline, gamma aminobutyric acid (GABA), and Glutamate levels in the cerebral cortex of mice brains were measured with ELISA, using a commercial kit (ImmuSmol, Inc., France). Briefly, one side of cerebral cortex was isolated from brain and homogenized in RIPA buffer with protease inhibitor (Santa Cruz Biotechnology, Japan). After centrifugation (10,000 x g, 30 min), 50 μL supernatant (protein samples) or 10 μL standards were used for catecholamine extraction, acylation and determination according to manufacturer’s instructions. After optimal color development, the reaction was stopped and the absorbance at 450 nm was recorded. Dopamine, noradrenaline, GABA, and glutamate levels were computed by correcting for the protein concentration. Protein estimation was conducted using 2-D quant kit (GE Healthcare Inc., Japan) according to procedure of the kit and the data were expressed as ng/μg protein.

### Statistical analysis

Data from quantification was collated into Microsoft Excel. Statistical analysis was performed using GraphPad Prism 6. When more than one treatment group were compared statistical analysis were preformed using one-way ANOVA followed by Bonferroni′s post hoc test. Also, Statistical analysis of the results obtained from MWM was carried out using two-way ANOVA with Ryan-Einot-Gabriel-Welsch multiple range test. A Student′s t test was used when only one group was compared with the control. All *in vitro* experiments were done in three or more biological replicates. Differences was considered significant at values of P < 0.05 * or P < 0.01**.
